# The Evaluation of the Clinical, Laboratory, and Radiological Findings of 16 Cases of Brucellar Spondylitis

**DOI:** 10.1155/2016/8903635

**Published:** 2016-09-08

**Authors:** Baohui Yang, Hongbo Hu, Jie Chen, Xijing He, Haopeng Li

**Affiliations:** Department of Orthopaedics, Second Affiliated Hospital of Xi'an Jiaotong University, Xi'an 710004, China

## Abstract

*Objective*. To evaluate the clinical, laboratory, and radiological presentation of 16 cases of brucellar spondylitis.* Methods*. The clinical manifestations, laboratory tests, and imaging findings of 16 patients (aged from 24 to 66 years) with brucellar spondylitis treated between September 2012 and September 2014 at the Second Affiliated Hospital of Xi'an Jiaotong University (Xi'an, China) were retrospectively analyzed.* Results*. Clinical manifestations included high fever, severe pain, sweating, and fatigue. One patient had epididymitis, and two showed clear signs of spinal nerve damage. Laboratory tests showed elevated erythrocyte sedimentation rate (ESR) and C-reactive protein content. Serum brucella agglutination tests were positive, and 11 brucella blood cultures were positive. Imaging manifestations mainly consisted of abnormal signals in the intervertebral space or abnormal signals in the adjacent vertebral bodies (16/16, 100%) in magnetic resonance imaging (MRI), disc space narrowing (14/16, 88%) in X-ray and MRI, or bone destruction and sclerosis around the damaged zone (13/16, 81%) in computed tomography, with rare cases of psoas abscess (2/16, 13%) and sequestrum (1/16, 6%).* Conclusion*. Since brucellar spondylitis exhibited characteristic clinical and imaging manifestations, it could be diagnosed with specific laboratory tests. Early MRI examination of suspected cases could improve rapid diagnosis.

## 1. Introduction

The zoonosis brucellosis is most often caused by the consumption of milk or meat contaminated with brucella. This zoonosis is distributed worldwide and represents an important public health problem in China. Previously, brucellosis was considered a rare disease; however, in recent years it has increased in incidence. In the musculoskeletal system, brucella most often invades the spine [1], particularly the lumbar spine and thoracolumbar segment. As the imaging characteristics of brucella infection of the spine are similar to those of spondylitis diseases, such as tuberculosis, it is often misdiagnosed. Between September 2012 and September 2014, a total of 16 patients with brucellar spondylitis were treated in our department. Here we report the clinical, laboratory, and radiological findings.

## 2. Methods

### 2.1. Study Design

This study was developed in accordance with the Declaration of Helsinki's code of ethics and approved by the Second Affiliated Hospital of Xi'an Jiaotong University (Xi'an, China). All participants provided written informed consent. Thirteen males and 3 females (19%), aged 24–66 years, were enrolled. Five patients were from Gansu, 2 from Shaanxi, 4 from Ningxia, 3 from Inner Mongolia, and 2 from Shanxi. Thirteen had a history of exposure to cattle and sheep, 1 had been on a tour of Inner Mongolia four months prior, and another 2 were uncertain about whether they had a history of animal exposure. Disease onset occurred in the lumbar spine in 14 cases, the thoracic spine in 1 case, and the cervical spine in 1 case. The time from onset to diagnosis was 14–45 days, and most patients had visited multiple hospitals.

### 2.2. Study Methodology

In addition to the two patients who had been diagnosed with brucella spondylitis, all patients admitted to hospital provided medical history and underwent clinical examination and laboratory tests including measurements of erythrocyte sedimentation rate (ESR), C-reactive protein, serum agglutination, and blood culture for* Brucella*, as well as purified protein derivative test, T-SPOT, and other tests performed in order to exclude tuberculosis. All patients also underwent X-ray, computed tomography (CT), and magnetic resonance imaging (MRI) examination, and conclusive diagnosis was made according to the evaluation of the clinical, laboratory, and radiological findings.

## 3. Results

### 3.1. Clinical Symptoms and Signs

Acute onset was reported in 14 cases, the main symptoms of which were afternoon fever, back pain, fatigue, and sweating. In five cases, lower back pain occurred first, followed by fever with aggravated back pain after 1-2 weeks. In one case, fever and epididymis pain occurred first, and the patient was diagnosed with epididymitis in the urology department. However, the patient was diagnosed with brucellar spondylitis one week later after reporting neck pain. Fever often occurred in the afternoon, exceeding 38.5°C, and was accompanied by sweating and fatigue but resolved spontaneously in some patients. Most patients exhibited remittent fever. Physical examination revealed pain with deep percussion that occurred at the site of the lesion and limited mobility of the spine. Two cases were diagnosed at local epidemic prevention departments and were transferred to our hospital due to symptoms of nerve damage, and the preoperative Frankel grades defining the degree of neurological involvement [2] for these cases were C and D.

### 3.2. Laboratory Tests

Laboratory tests performed at admission revealed ESRs ranging from 28 to 94 mm/h (average 44 mm/h) and C-reactive protein levels ranging from 7.4 to 21 mg/dL (average 19 mg/dL). With the exception of the two cases whose diagnosis was confirmed at the local epidemic prevention departments, all patients underwent serum agglutination testing for tuberculosis and blood culture testing for brucellosis within three days of admission. Rose Bengal plate tests for brucellosis were positive, and 12 patients had brucella serum agglutination test results over 1 : 160 and 11 patients' blood cultures were positive for brucellosis.

### 3.3. Imaging Findings

**Figure 1:**
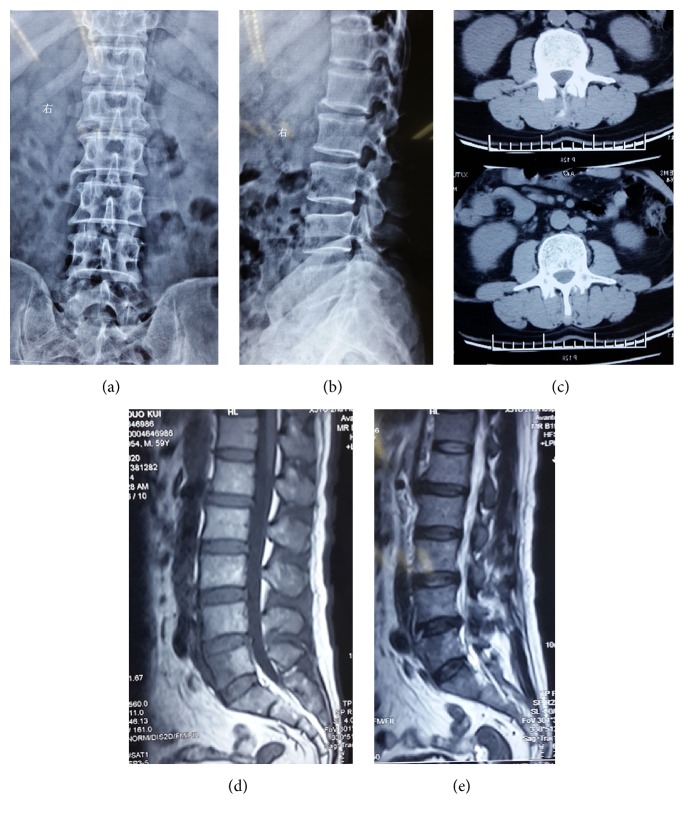
A 59-year-old male sustained fever and low back pain for 2 weeks. The anteroposterior and lateral plain film and lumbar CT scan were normal, but the MRI revealed a change in intensity at L5.

**Figure 2:**
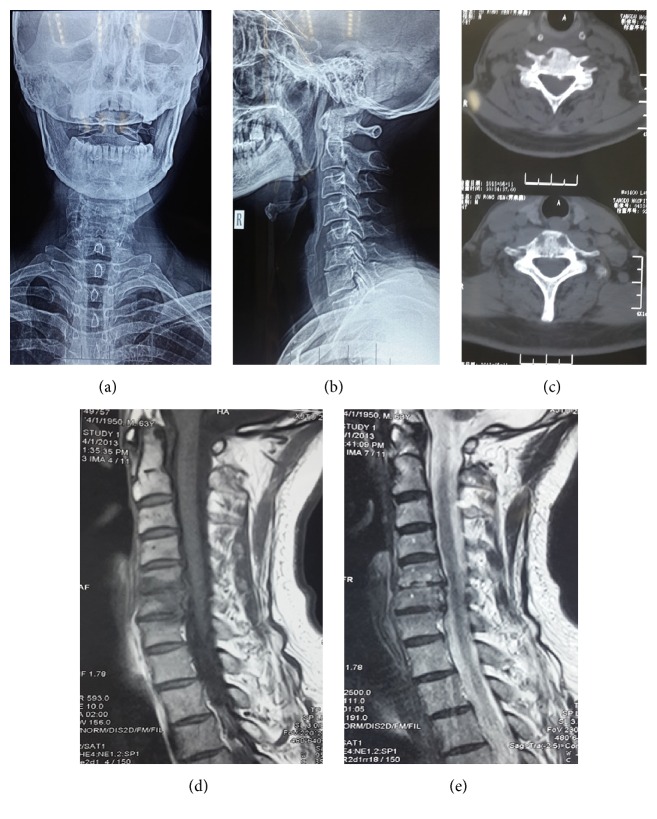
A 65-year-old male sustained fever and testicular pain for 1 week. After receiving treatment at the urinary surgery department for 1 week, the symptoms were relieved, but cervical pain emerged after another week. The anteroposterior and lateral plain of the cervical spine revealed narrow intervertebral spaces of C5-6 and C6-7. The CT scan revealed destruction of the cortical and cancellous bone. The edges of the centrums were sclerotic, but sequestrum was not found. T1 and T2 MRI revealed that the intensity of the C5-6 was abnormal and that the intervertebral space was narrow. The brucella serum agglutination test result exceeded 1 : 160. Brucella blood culture was negative. The patient was diagnosed with brucella infection.

**Figure 3:**
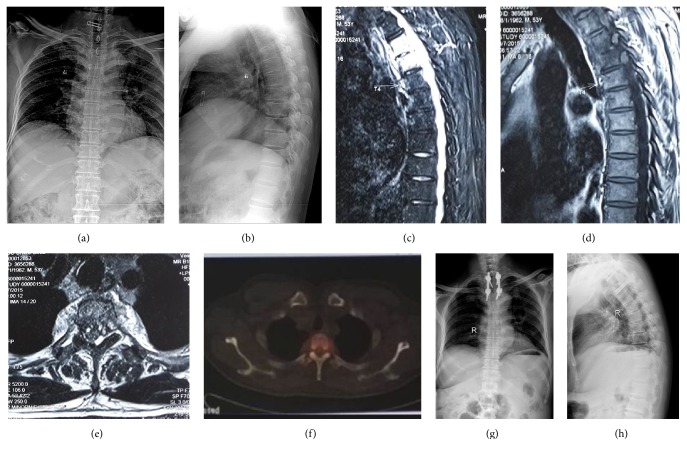
4 months after brucella infection. An anteroposterior and lateral plain film of a 53-year-old man revealed a narrowed T2-3 intervertebral space. The MRI showed that the soft tissues in front of and near the centrum and inside the canalis spinalis were swollen and abscessed. The corresponding segment of the spinal cord was compressed. Before performing the debridement and fusion with internal fixation, the Frankel grade was C. 2 months after the surgery the Frankel grade was E.

All patients had a complete record of X-ray, CT scan, and MRI examination. Simultaneous radionuclide bone scans were performed for three cases. No obvious abnormalities were found in the early X-rays and even CTs of most patients. However, involvement of the vertebral disc space, along with a low T1-weighted imaging (T1WI) signal and a high T2WI signal above and below the vertebral body, was apparent in early MRIs (Case 1, Figure 1); obvious intervertebral space stenosis and vertebral destruction were observed at the mid-advanced and advanced stage, with sclerosis surrounding the lesion, but sequestrum was rare (Case 2, Figure 2). At an advanced stage, where soft tissue swelling or spinal cord compression was observed, the enhanced CT showed significant radionuclide concentration (Case 3, Figure 3). The MRIs of the 16 cases that were diagnosed with brucella spondylitis all exhibited a signal abnormality in the disc or adjacent vertebral body, while only 2 cases (13%) had paraspinal abscess which were limited without obvious “streamer” performance. Disc space narrowing was observed in 14 cases (88%) by X-ray or MRI scanning. What is more, bone destruction and sclerosis were observed around the damaged zone in 14 patients (81%) and sequestrum formation was observed in 1 case (6%) by CT scan.

## 4. Discussion

Brucellosis is a zoonotic infectious disease and was first described by Kulowski and Vinke [3] in 1932. The incidence of brucellar spondylitis ranges worldwide from 2% to 53% [4], and in recent years, with the development of China's animal husbandry and tourism industries, the incidence of this disease in China is increasing, and orthopedic physicians should be informed of its appearance and treatment.

### 4.1. Clinical Manifestations and Signs of Brucellar Spondylitis

Typical manifestations of brucellar spondylitis can be summarized as a triad of back pain, high fever in the afternoon, and sweating, with signs of infection in the intervertebral space and vertebral bodies [5]. Back or neck and back pain are often severe. In this study, 15 patients complained of severe back pain at the early stage of disease onset; this pain was often unbearable and required oral pain medication. Only one patient with cervical disease in this study had pain that was manifested as a tolerable dull pain at the early stage, likely because the epididymitis pain masked the extent of their neck pain, and the weight borne by the cervical spine was relatively light. Almost all patients experienced fever, mostly manifested as high fever in the afternoon, often with sweating after the high fever broke. The fevers were mostly remittent, at 38.5°C or higher, and spontaneously resolved in some patients. Due to the impact of the lower back pain or neck and back pain, the patients' spinal mobility levels were reduced. Clinical manifestations included pain with deep percussion at the lesion site and limited spinal mobility. In cases of spinal cord compression, abnormal limb sensation, muscle strength, and reflection would occur. In this study, two cases experienced spinal cord compression: in one patient at the thoracic spine (preoperative Frankel grade C) and in the other at the lumbar spine (preoperative Frankel grade C).

### 4.2. Imaging of Brucellar Spondylitis

After examining the MRIs of patients with brucellar spondylitis and comparing them to X-ray and CT results, Bozgeyik et al. indicated that MRI is the preferred imaging method for the diagnosis of brucellar spondylitis [6]. In this study, X-ray examinations were performed at local hospitals for five patients in the early stage, due to lower back pain and fever, and the reports revealed no obvious abnormalities. In contrast, the MRIs performed at our hospital revealed signal abnormalities in the intervertebral space and the vertebral bodies. This phenomenon could be attributed to the lack of awareness of this disease and the limited ability of X-ray to detect hallmarks of this disease's acute phase. In the acute phase, the MRIs revealed low T1WI signals in the vertebral lesion, endplate, and disc, with a high T2WI signal. In the subacute and chronic phases the invaded vertebral bodies and intervertebral discs exhibited heterogeneous T1WI and T2WI signals. Thus, MRI is a more sensitive tool with which to recognize early bone infection, and it should be considered as a method of early diagnosis [7, 8]. Although Akman et al. did not observe paraspinal soft tissue abscess in brucellar spondylitis [9], in recent years, further reports have described conflicting observations. In this study MRIs of the two patients who underwent surgery revealed thin and irregular enhancements of paraspinal abscess walls and ill-defined soft tissue edema signals (Case 3), but these patients' abscesses were not manifested in a typical manner, such as the cold abscesses of tuberculosis. In the acute phase, X-ray or CT might not reveal any obvious abnormalities, but in the chronic phase, bone destruction and proliferation are more noticeable, and vertebral bone hyperplasia, bone destruction, and sclerosis around the lesion were often observed, while sequestrum was rare. In this study, sequestrum was observed in only one case. Overall, the typical imaging characteristics of brucella spondylitis are disc space narrowing, signal abnormities of the vertebral body and disc, bone destruction, and hardening, and signs of sequestrum formation and limited paraspinal abscess are rare. In recent years, studies have suggested that in patients with brucellar spondylodiskitis F-18 FDG PET/CT scan can provide more information on the spread of the infection compared to MRI [10]; however, the cost of MRI is prohibitively high for routine clinical application.

### 4.3. Laboratory Tests for Brucellar Spondylitis

Most scholars believe that blood culture is the gold standard for the diagnosis of brucellar spondylitis. However, in clinical practice, brucellosis culture requires a long incubation period and specific conditions. In addition, brucellosis culture does not always produce a positive bacterial culture, making it unsuitable to serve as a conventional detection method. Positive bacterial cultures were obtained for only 11 of the 16 patients with confirmed brucellar spondylitis. Therefore, we recommend that brucellosis is diagnosed according to clinical manifestations and the isolation of* Brucella* species from blood or bone marrow cultures [11, 12]. In the absence of bacteriologic confirmation, positive serology for brucella (titer over 1 : 160 in a standard tube agglutination test or a 4-fold increase in the brucella-antibody titer) is needed for definite diagnosis [12]. Increases in ESR and C-reactive protein levels would also occur in these patients. In this study, the ESR ranged from 28 to 94 mm/h (average 44 mm/h), and the C-reactive protein levels ranged from 7.4 to 21 mg/dL (average 19 mg/dL). However, increased ESR has been observed in a majority of case reports, which may represent a useful measure for assessing the response to therapy [13].

### 4.4. Differential Diagnosis

Brucellar spondylitis often needs to be differentiated from ordinary discitis, vertebral osteomyelitis, tumors, and most importantly spinal tuberculosis. Spinal tuberculosis is often manifested as mild back pain, and fever was rare; it was associated mainly with vertebral body destruction, with rare sclerosis. Sequestrum was common, while paraspinal “cold abscess” was generally observed. Of course, the ultimate method of identification may still require confirmation with a combination of laboratory and pathology tests.

### 4.5. Treatment for Brucellar Spondylitis

In the acute phase when no obvious damage to the vertebral bodies is observed, patients with spinal cord, nerve damage, and abscesses achieved good therapeutic outcomes with drug treatment. In this study, 14 patients received conservative drug treatment consisting of rifampin 600–900 mg/d and hydrochloride polyene doxycycline 200 mg/d with the morning meal [13, 14]. Additional streptomycin or tetracycline treatments have been reported in the literature. This type of brucellosis has been reported to be caused by intracellular bacteria and is associated with a relatively high relapse rate [11]; however, follow-up at 12 months revealed no recurrence.

A few patients may require surgical treatment [15]. Spinal surgery is considered in cases of spinal instability, cord compression, radiculopathy, cauda equina syndrome, and epidural abscess formation [16, 17]. Either anterior or posterior surgery can be selected. Posterior surgical approaches were applied in two cases in this study, and both achieved good surgical results. The Frankel grade of one case was increased from preoperative grade D to postoperative grade E (after two months), and the Frankel grade of the other case was increased from preoperative grade C to postoperative grade E (after two months).

In summary, due to its low incidence, lack of standard diagnosis procedure and treatment regimen, and significant regional variation, some physicians may not be equipped to recognize and treat brucellar spondylitis. This lack of information may delay treatment. However, in this study we found that the clinical and imaging manifestations of brucellar spondylitis shared characteristic features and that brucellar spondylitis could be diagnosed with specific laboratory tests. Our results indicate that early MRI examination of suspected cases could improve rapid diagnosis.
